# How the cerebellum learns to build a sequence

**DOI:** 10.7554/eLife.40660

**Published:** 2018-08-23

**Authors:** Reza Shadmehr

**Affiliations:** Laboratory for Computational Motor Control, Department of Biomedical EngineeringJohns Hopkins School of MedicineBaltimoreUnited States

**Keywords:** learning, sequence learning, cerebellum, rabbit, Other

## Abstract

Rabbits can learn the biological analogue of a simple recursive function by relying only on the neurons of the cerebellum.

**Related research article** Khilkevich A, Zambrano J, Richards MM, Mauk MD. 2018. Cerebellar implementation of movement sequences through feedback. *eLife* **7**:e37443. doi: 10.7554/eLife.37443

Sequential patterns like the Fibonacci numbers, as well as the movements that produce a tied shoelace, are examples of recursion: the process begins with a seed that a system uses to generate an output. That output is then fed back to the system as a self-generated input, which in turn becomes a new output. The result is a recursive function that uses a seed (external input) at time *t* to generate outputs at times *t*, *t + D*, *t* + 2*D* and so on (where *D* is a constant interval of time). Now, in eLife, Andrei Khilkevich, Juan Zambrano, Molly-Marie Richards and Michael Mauk of the University of Texas at Austin report the results of experiments on rabbits which shed light on how the brain learns the biological analogue of a recursive function (Khilkevich et al., 2018).

To present the seed that started a sequence of motor outputs, Khilkevich et al. electrically stimulated the mossy fibers that provided inputs to the cerebellum. Near the end of the period of mossy fiber stimulation, they electrically stimulated the skin near the eyelid, which caused the rabbits to blink. The blink was the motor output. With repeated exposure to the mossy fiber input and the eyelid stimulation, the cerebellum learned to predict that the mossy fiber stimulation would be followed by the eyelid stimulation (Krupa et al., 1993), which then led to an anticipatory blink at time *t*. That is, given an input to the cerebellum at time *t*, the animals learned to produce an output at the same time. The technical term for this kind of learning is classical conditioning.

However, the goal for the rabbits was to learn to blink not just at time *t*, but also at times *t + D*, *t* + 2*D* and so on. That is, the challenge for the animal was to learn to use its own motor output at time *t* (the eye blink) as the cue needed to produce a second blink at *t + D*. To do this, Khilkevich et al. measured the eyelid response at time *t*. If the eye was less than 50% closed, they stimulated the eyelid as usual. However, if the eye was more than 50% closed, they stimulated it 600 milliseconds later (that is, at *t + D*). The critical point is that there was no input to the mossy fibers at *t + D*. Although earlier experiments had shown that the cerebellum was not able to associate a mossy fiber input with stimulation of the eyelid when the delay between them was longer than 400 milliseconds (Kalmbach et al., 2010), Khilkevich et al. found that the animals learned to blink not only at time *t*, but also at time *t + D*.

Their hypothesis was that the sequence was learned through recursion: a copy of the commands for the blink at *t* was sent as input to the cerebellum, allowing it to associate these commands with the eyelid stimulation at *t + D*, and thereby learning to blink at *t + D*. An elegant experiment confirmed this hypothesis: Khilkevich et al. found that after training was completed and the rabbits blinked at times *t* and *t + D*, omitting the eyelid stimulation at time *t* resulted in the extinction of the blinks at times *t* and *t + D*. Moreover, and rather remarkably, even if the eyelid was subsequently stimulated at time *t + D*, there was still no blink. This established the fundamental feature of the recursive function: without the blink at time *t*, which was generated because of the mossy fiber input at *t*, the animal could not produce a blink at time *t + D*.

Under normal conditions, the principal cells of the cerebellum, Purkinje cells, produce a steady stream of simple spikes. As the animal learns to associate the mossy fiber input with the eyelid stimulation, the Purkinje cells reduce their simple spike discharge just before the blink at time *t*, and again before the second blink at *t + D* (Jirenhed et al., 2017). Khilkevich et al. found that the modulation of the spikes before *t + D* appeared to be causal, because there was no blink response at *t + D* if there was no modulation around time *t + D*. The timing of the modulation at *t* and *t + D* also appeared consistent with a role for the cerebellum in generating the recursive function.

The results of Khilkevich and co-workers expand the range of learning behaviors that have been ascribed to the cerebellum. Earlier work had shown that Purkinje cells learn to associate motor commands with their sensory consequences (Herzfeld et al., 2015), forming 'forward models' that enable animals to control their movements with precision and accuracy (Heiney et al., 2014; Herzfeld et al., 2018). The new results demonstrate that Purkinje cells can also learn recursive functions, using a seed plus feedback from the animal’s own actions to construct a sequence of movements.

## References

[bib1] Heiney SA, Kim J, Augustine GJ, Medina JF (2014). Precise control of movement kinematics by optogenetic inhibition of Purkinje cell activity. Journal of Neuroscience.

[bib2] Herzfeld DJ, Kojima Y, Soetedjo R, Shadmehr R (2015). Encoding of action by the Purkinje cells of the cerebellum. Nature.

[bib3] Herzfeld DJ, Kojima Y, Soetedjo R, Shadmehr R (2018). Encoding of error and learning to correct that error by the Purkinje cells of the cerebellum. Nature Neuroscience.

[bib4] Jirenhed DA, Rasmussen A, Johansson F, Hesslow G (2017). Learned response sequences in cerebellar Purkinje cells. PNAS.

[bib5] Kalmbach BE, Ohyama T, Mauk MD (2010). Temporal patterns of inputs to cerebellum necessary and sufficient for trace eyelid conditioning. Journal of Neurophysiology.

[bib6] Khilkevich A, Zambrano J, Richards MM, Mauk MD (2018). Cerebellar implementation of movement sequences through feedback. eLife.

[bib7] Krupa DJ, Thompson JK, Thompson RF (1993). Localization of a memory trace in the mammalian brain. Science.

